# Association of clinical findings in Yusho patients with serum concentrations of polychlorinated biphenyls, polychlorinated quarterphenyls and 2,3,4,7,8-pentachlorodibenzofuran more than 30 years after the poisoning event

**DOI:** 10.1186/1476-069X-7-47

**Published:** 2008-10-02

**Authors:** Yoshiyuki Kanagawa, Shinya Matsumoto, Soichi Koike, Bunichi Tajima, Noriko Fukiwake, Satoko Shibata, Hiroshi Uchi, Masutaka Furue, Tomoaki Imamura

**Affiliations:** 1 Department of Planning Information and Management, The University of Tokyo Hospital, Tokyo, Japan; 2 Teradata Division, NCR JAPAN Ltd, Tokyo, Japan; 3 Department of Dermatology, Graduate School of Medical Sciences, Kyushu University, Fukuoka, Japan; 4 Department of Public Health, Health Management and Policy, Nara Medical University, School of Medicine, Nara, Japan

## Abstract

**Background:**

The Yusho poisoning incident, which was caused by rice bran oil contaminated with polychlorinated biphenyls (PCBs), polychlorinated quarterphenyls (PCQs) and polychlorinated dibenzofurans (PCDFs) generated by heat denaturation of PCB, occurred in 1968 in western Japan. Annual physical, dermatological, dental, ophthalmological and laboratory examinations were conducted for Yusho patients after the incident. From 2001, blood levels of individual PCDF congeners were also measured. The blood levels of 2,3,4,7,8-pentachlorodibenzofuran (2,3,4,7,8-PeCDF), PCBs and PCQs in Yusho patients were found to be significantly higher than those of the general population. We investigated the relationships between blood concentrations of 2,3,4,7,8-PeCDF, PCBs and PCQs in Yusho patients and the items measured in the annual medical examination.

**Methods:**

Medical and laboratory examination data from 501 Yusho patients enrolled in the study from 2001 to 2004 were analyzed. The relationships between blood 2,3,4,7,8-PeCDF, PCB and PCQ concentrations and medical/laboratory examination data were investigated using principal components and logistic regression analyses.

**Results:**

Serum Concentrations of 2,3,4,7,8-PeCDF, PCBs and PCQs in blood tended to correlate with either acneform eruptions, black comedones, cutaneous and mucosal pigmentation, and hypersecretion of meibomian glands as well as general fatigue, headaches, cough/sputum, abdominal pain, arthralgia, increased blood sugar, increased serum γ-GTP and decreased total bilirubin. The majority of these signs and symptoms are included in the diagnostic criteria for Yusho.

**Conclusion:**

After Yusho patients had suffered chronic exposure to these chlorinated compounds for more than 35 years, the serum concentration of 2,3,4,7,8-PeCDF in blood was significantly related to arthralgia and decreased albumin/globulin (A/G) ratio; the serum concentration of PCBs was significantly related to ophthalmologic symptoms; and the serum concentration of PCQ to increased total cholesterol. These findings suggest that the co-contaminants may affect other functions than those originally associated with Yusho.

## Background

Yusho was a food poisoning incident that occurred in western Japan in 1968 [1-8]. When first reported, the food poisoning incident known as Yusho was considered to be caused by polychlorinated biphenyls (PCBs). However, following a number of studies, it is now considered to be caused by complex poisoning with polychlorinated quarterphenyls (PCQs) and polychlorinated dibenzofurans (PCDFs) [3-6]. Thirty-seven years have passed since the Yusho incident occurred, and more than 1,800 patients are known to have been affected.

Yusho patients are known to present with various symptoms related to the skin, eyes and teeth, and have abnormal findings on physical examinations [8-14]. The severity of symptoms in Yusho patients has gradually improved over the past 37 years. However, a number of patients still suffer from specific Yusho symptoms [3,4,8]. The initial diagnostic criteria published in 1968 were mainly: 1) proven history of ingestion of contaminated rice bran oil; 2) prominent dermatological, ophthalmological and mucosal signs; and 3) several nonspecific general signs and symptoms. Hyperglyceridemia, pulmonary disorders, intractable headache, elevated blood PCB concentrations and specific PCB patterns on gas chromatography were added to the initial diagnostic criteria in 1972 and 1976. Blood PCQ concentrations were added to the criteria in 1981 [3].

With recent advances in techniques for measuring individual PCDF congeners, it has become possible to precisely measure 2,3,4,7,8-pentachlorodibenzofuran (2,3,4,7,8-PeCDF) blood concentrations using as little as 5 ml of blood [17,18]. Thus, measurements of 2,3,4,7,8-PeCDF blood concentrations have been initiated since 2001 in the routine mass screening of Yusho patients. The mean blood concentrations of PeCDF in these patients have been shown to be more than 10 times higher than those in normal controls [3]. In 2004, the blood 2,3,4,7,8-PeCDF concentration was added to the present diagnostic criteria (Table 1).

**Table 1:** Diagnostic criteria for Yusho (updated)

The diagnostic criteria for Yusho were revised on October 26, 1972; supplemented on June 14, 1976; and an item related to blood polychlorinated quarterphenyl (PCQ) level was added on June 16, 1981. The study group of Yusho started to measure blood levels of dioxins in annual medical check-ups from 2001. It was considered appropriate to add an item corresponding to the blood 2,3,4,7,8-PeCDF level; therefore the criteria were supplemented and further revised on September 29, 2004.

**Conditions of the incident**
1. Proof that Kanemi rice bran oil contaminated with polychlorinated biphenyls (PCBs) was ingested.
2. There are also some cases in which PCB is transferred from mothers with Yusho to their children.
3. Familial occurrence is also seen in many cases.

**Important manifestations**
1. Acneform eruptions Black comedones* seen on the face, buttocks and other intertriginous sites; comedones with inflammatory manifestations; and subcutaneous cysts with atheroma-like contents that tended to suppurate.
2. Pigmentation Pigmentation of the face, palpebral conjunctivae, gingivae**, and nails etc. (including so-called 'black babies').
3. Hypersecretion of the meibomian glands.
4. Unusual composition and concentration of PCBs in the blood.
5. Abnormal level of blood PCQ
(1) ≥ 0.1 ppb: an abnormally high concentration.
(2) 0.03 to 0.09 ppb: the boundary between high and normal concentrations.
(3) ≤ 0.02 ppb (detection limit): normal concentration.
6. Abnormal level of blood PeCDF
(1) ≥ 50 pg/g lipids: an abnormally high concentration.
(2) 30 to 50 pg/g lipids: a relatively high concentration.
(3) < 30 pg/g lipids: normal concentration.

**Standard symptoms and findings**
1. Subjective symptoms
(1) General fatigue
(2) Headaches, dull headaches
(3) Paresthesia of the extremities (abnormal sensation)(4) Increased eye discharge
(5) Cough and sputum
(6) Inconstant abdominal pain
(7) Altered menstruation
2. Objective findings
(1) Manifestation of bronchitis
(2) Deformation of nails
(3) Bursitis
(4) Increased neutral fat in the serum
(5) Increased serum γ-glutamyl transpeptidase (γ-GTP)
(6) Decrease in serum bilirubin
(7) Small-for-date baby
(8) Growth retardation and dental abnormality (retarded eruption of permanent teeth)

* Black comedones (other sites):

black comedones appearing on body parts other than the face, auricle, and trunk

** Palatal findings

Palatal lesions known to occur in Yusho patients include pigmentation and parakeratosis in the gingiva, dental root dysplasia, and odontatrophia.

Palatal findings from examinations in Yusho patients are recorded as follows:

(1) The upper teeth and lower teeth are each divided into 3 sites:

site 1 = 7–4, site 2 = 3-3, and site 3 = 4–7.

(2) Pigmentation patterns are divided into the following patterns recognized in Yusho patients:

Condition 1 = diffuse, Condition 2 = punctate, Condition 3 = linear,

Condition 4 = zonal, Condition 5 = cloudy, Condition 6 = island shaped

In this study, we analyzed the results of medical examinations of Yusho patients whose blood 2,3,4,7,8-PeCDF concentrations were measured from 2001 to 2004 (33 to 37 years after the occurrence of the Yusho disaster), and investigated the relationships among the 2,3,4,7,8-PeCDF, PCB and PCQ blood concentrations and the clinical data from physical and laboratory examinations.

## Methods

### Subjects and medical check items

Since immediately after the incident occurred, the Yusho Study Group has conducted annual health checks of Yusho patients. Between 2001 and 2004, a total of 501 individuals (81 individuals in 2001, 371 in 2002, 343 in 2003 and 292 in 2004, including multiple health checks) underwent the Yusho mass screening. In addition to blood PeCDFs, PCBs and PCQs serum concentrations, 241 check items (52 items in a questionnaire, 55 physical and laboratory examinations, 21 dermatological examinations, 108 dental examinations, and 5 ophthalmological examinations) were carried out (Table 2).

**Table 2:** Annual medical check examination sheet of Yusho patients

**(1). Laboratory examination**
**Blood concentrations of PCB- and dioxin-related compounds**
Total PCB, Peak 1, Peak 2, Peak 3, PCB pattern, CB ratio, Total PCQ, Dioxin-related compounds
Urinalysis (Protein, Sugar, Occult blood, Urobilinogen, pH)
**Hematological examination**
ESR (1-hour), ESR (2-hour), WBC, RBC, Hb, Ht, MCV, MCH, MCHC, PLT
**Blood chemistry**
T-Bil, D-Bil, GOT, GPT, TP, Alb, albumin/globulin(A/G) ratio, ZTT, TTT, ALP, LAP, γ-GTP, ChE, LDH,
CPK, TC, HDL-chol, TG, β-lip, BUN, Cre, Na, K, Ca, P, Amy, blood sugar level
**Immunological examination **(HBs antigen, α-fetoprotein)

**(2). Interview and physical examination**
**Life history **(Alcohol, Smoking)
**Chief complaint**
**Past history**(Before the incident, After the incident)
**Subjective symptoms**
General fatigue, Headache, Cough, Sputum, Abdominal pain, Diarrhea, Constipation
Numbness, Arthralgia, menstruation disorders
**Physical examination**
Body height, Body weight, Heart rate, Blood pressure, Nutrition, Heart sounds, Respiratory sounds,
Chest radiography, ECG, Abdominal ultrasonography,
Hepatomegaly, Splenomegaly, Edema, Lymphadenopathy, Tendon reflex, Sensory examination,

**(3). Dermatological examination**
**Interview**
Recent tendency to purulent skin eruptions, Recent recurrence of cystic lesions,
Past history of acneform eruptions, Past history of pigmentation,
**Physical examination **(severity and sites)
Black comedones, Acneform eruptions, Scar formation, Pigmentation, Nail deformity,

**(4). Dental examination**
**Chief complaint**
Toothache, Gingival bleeding, Pus discharge, Gingival swelling, Feeling of tooth extrusion, Pigmentation
**Items for oral examination **(No/Yes, site)
Gingivitis, Marginal periodontitis, Retarded eruptions of permanent teeth,
Tooth pigmentation, Odontogenesis imperfecta, Abnormal occlusion, Other findings,
**Mucosal pigmentation **(severity, site, *pattern, **color)
Upper gingivae, Lower gingivae, Rt. buccal mucosa, Lt. buccal mucosa, Palate,
Upper lip, Lower lip
**Teeth radiograph **(No/Yes)
*Selection items for pattern (Diffuse, Spotted, Band-like, Linear, Faint, Scattered)
**Selection items for color (Black, Brownish, Dark-brownish)

**(5). Ophthalmological examination**
**Subjective symptoms **(Abnormal discharge from the eyes)
**Objective symptoms**
Edema of the eyelid, Conjunctival pigmentation, Cysts of meibomian glands,
Cheesy secretion from meibomian glands,

### Statistical analysis

The relationships between blood 2,3,4,7,8-PeCDF concentrations(serum) and the physical/laboratory test items were analyzed using logistic regression analysis. Since the serum half-life of 2,3,4,7,8-PeCDF is long and the present blood concentrations are well correlated with the amount of exposure at the time of the incident, the correlations with these was examined [19]. Logistic regression analysis uses a formula to relate several explanatory variables to objective ones (2 values). We used the following equation which included results [*y*] and several factors [*x*_1_, *x*_2_, ..., *x*_n_] affecting these results with *β*_i _as coefficients

*g*(*x*) = *β*_0_+*β*_1_*x*_1_+*β*_2_*x*_2_+ ...+*β*_n_*x*_n_

*y *= *e*^*g*(*x*)^/(*e*^*g*(*x*)^+1)

To conduct a logistic regression analysis, we conducted a principal component analysis as an auxiliary analysis to decide the explanatory variables. Specifically, of the 241 items examined in the Yusho medical checkup, the principal component analysis was conducted on 172 items, except for those related to frequency. As a result, examination items with 1 or higher eigenvalues and high factor scores in the principal component analysis were used as representative variables. In deciding the representative variables, items with high factor scores were not selected mechanically, but the following criteria were considered:

(1) Items included in the criteria.

(2) Items considered to be medically significant.

(3) A weak factor representing an item selected by multiple factors.

Furthermore, we confirmed that items whose associations with Yusho have been indicated were not overlooked, by reference to the criteria. We extracted 49 items, including 13 questionnaire-related items, 11 physical and laboratory examination items, 10 dermatological examination items, 12 dental examination items and 3 ophthalmological examination items, as representative variables. (Table 3)

**Table 3:** Variables selected for principal components analysis

**No.**	**Variables**	**Factor Score**	**Examination classification**
1	Pigmentation of lower gingivae	0.735	Dental examination
2	Blood sugar (increase)	0.443	Laboratory examination
3	Abdominal pain	0.408	Questionnaire
4	Past history of pigmentation	0.498	Dermatological examination
5	Arthralgia	0.437	Questionnaire
6	Pigmentation of the upper lip (diffuse)	0.401	Dental examination
7	Triacylglycerol (increase)	0.361	Laboratory examination
8	Sputum	0.307	Questionnaire
9	Mean corpuscular volume (increase)	0.419	Laboratory examination
10	γ-GTP (increase)	0.367	Laboratory examination
11	Pigmentation of the upper lip (band-like)	0.581	Dental examination
12	albumin/globulin(A/G) ratio (decrease)	-0.471	Laboratory examination
13	General fatigue	-0.327	Questionnaire
14	Pigmentation (toe nails)	0.447	Dermatological examination
15	Tooth pigmentation	0.336	Dental examination
16	Pigmentation of the palatal mucosa	0.204	Dental examination
17	Pigmentation of the right buccal mucosa (band-like)	0.339	Dental examination
18	Past history (after the incident)	0.241	Questionnaire
19	Dental chief complaint	0.389	Dental examination
20	Nail deformity	-0.272	Dermatological examination
21	Numbness	0.208	Questionnaire
22	Pigmentation (face)	0.280	Dermatological examination
23	Abnormal discharge from the eyes	-0.297	Ophthalmological examination
24	Abnormal respiratory sounds	0.295	Physical examination
25	Pigmentation (left. buccal mucosa)	0.266	Dental examination
26	Total cholesterol (increase)	0.246	Laboratory examination
27	Cough	0.192	Questionnaire
28	Past history (Before the incident)	0.246	Questionnaire
29	Acneform eruptions (other sites)	0.297	Dermatological examination
30	Mucosal pigmentation of upper gingivae (linear)	0.363	Dental examination
31	Cheesy secretion from meibomian glands	-0.260	Ophthalmological examination
32	Presence of hepatomegaly	-0.317	Questionnaire
33	Direct-bilirubin (increase)	0.225	Laboratory examination
34	Abnormal heart sounds	0.239	Questionnaire
35	potassium level	0.286	Laboratory examination
36	Acneform eruptions (trunk)	0.216	Dermatological examination
37	Pigmentation (fingernails)	0.231	Dermatological examination
38	Malocclusion	0.274	Dental examination
39	Black comedones (other sites)	-0.323	Dermatological examination
40	Urinalysis protein	0.363	Laboratory examination
41	Systolic blood pressure (low)	-0.294	Questionnaire
42	Toothache	0.311	Dental examination
43	Edema of the eyelids	0.305	Ophthalmological examination
44	Headache	0.188	Questionnaire
45	Chief complaint	-0.224	Questionnaire
46	Pigmentation of the mucosa of the upper lip (spotted)	0.292	Dental examination
47	Black comedones (face)	0.240	Dermatological examination
48	Total bilirubin (increase)	0.225	Laboratory examination
49	Past history of acneform eruptions	0.248	Dermatological examination

Furthermore, the following patterns were set as objective variables for our logistic regression analyses:

#### 2,3,4,7,8-PeCDF blood concentration(serum)

2 categories: [≥ 50 pg/g lipids] and [< 50 pg/g lipids] (as indicated by the diagnostic criteria [3])

#### PCB blood concentration(serum)

2 categories: [≥ 2.0 ppb] and [< 2.0 ppb] (Categorized by median value)

#### PCQ blood concentration(serum)

2 categories: [≥ 0.10 ppb] and [< 0.10 ppb] (as indicated by the diagnostic criteria [3])

#### Other examination items

The 49 factors extracted by the principal component analysis were classified into normal and abnormal categories considering the characteristics of the data for each test item, from the following viewpoints:

(1) Factors for which the presence or absence of symptoms was confirmed by two steps in the medical checkup by a doctor were classified into two steps of presence or absence.

(2) Factors whose measurement results had normal value standards, such as blood test results, were classified into normal or abnormal.

(3) Items relevant to subjective symptoms, like sputum, arthralgia and general fatigue, were classified into "normal" or "abnormal" for each patient.

(4) Items evaluated into five grades (-, ±, +, ++ and +++) of symptoms, such as severity of pigmentation, were classified into two groups, based on the criterion of "+" or above, to determine the presence of symptoms.

To conduct analyses on the above 3 patterns, 2,3,4,7,8-PeCDF, PCB and PCQ blood concentrations were added to the explanatory variables. SPSS11.5J for Windows was used for the analyses.

## Results

2,3,4,7,8-PeCDF blood concentration as an objective variable, PCB and PCQ blood concentrations, blood glucose level, arthralgia, gender, total bilirubin, black comedones, acneform eruption, past history of skin pigmentation and acneform eruption, increased A/G ratio, abnormal respiratory sounds, blood potassium level, and total cholesterol showed less than a 0.05 level of significance. Most of these items are considered characteristic symptoms of Yusho. Even when PCB and PCQ blood concentrations are excluded from the explanatory variables, older age, A/G ratio, general fatigue, arthralgia, gender and oral pigmentation showed less than a 0.05 level of significance.

In contrast, when 49 factors were extracted by the principal component analysis (PCB and PCQ as objective variables, 2,3,4,7,8-PeCDF blood concentration as an explanatory variable), PCQ and PCB blood concentrations, arthralgia, presence or absence of previous history since 1968, A/G ratio and blood glucose level indicated a significance probability of ≤ 0.05 for the 2,3,4,7,8-PeCDF blood concentration. (Table 4)

**Table 4:** Results of logistic regression analysis(2,3,4,7,8-PeCDF blood level as an objective variable)

List of explanatory variables which showed less than 0.10 level of significance when 2,3,4,7,8-PeCDF blood level (2 categories) and the factors selected by the principal components analysis were used as objective variables and explanatory variables, respectively						
	Explanatory variables	β	Standard deviation	Wald	Significance probability	Exp (B)

1	PCB blood level	1.641	0.355	21.350	0.00000**	5.159
2	PCQ blood level	8.235	1.844	19.949	0.00001**	3771.361
3	Blood sugar (increase)	0.038	0.011	11.310	0.00077**	1.038
4	Arthralgia	3.734	1.159	10.382	0.00127**	41.857
5	Gender (female)	3.456	1.115	9.605	0.00194**	31.679
6	T-bilirubin (increase)	-3.310	1.194	7.681	0.00558**	0.037
7	Black comedones (face)	-2.216	0.836	7.021	0.00806**	0.109
8	Past history of skin pigmentation	3.576	1.435	6.209	0.01271**	35.735
9	A/G ratio (decrease)	1.978	0.825	5.748	0.01651**	7.225
10	Acneform eruptions (trunk)	3.809	1.650	5.331	0.02095**	45.088
11	Respiratory sounds (abnormal)	6.036	2.780	4.714	0.02991**	418.145
12	Acneform eruptions (other sites)	-5.514	2.721	4.107	0.04270**	0.004
13	Potassium level (increase)	-1.849	0.917	4.071	0.04361**	0.157
14	Past history of acneform eruptions	-2.630	1.304	4.065	0.04378**	0.072
15	Total cholesterol (increase)	-0.023	0.011	3.910	0.04799**	0.978
16	Heart sound (abnormal)	13.341	7.892	2.857	0.09096*	621883.792

List of explanatory variables which showed less than 0.10 level of significance when PCB and PCQ blood levels were excluded from the explanatory variables in the above analysis						

	Explanatory variables	β	Standard deviation	Wald	Significance probability	Exp (B)

1	Past history of pigmentation	1.719	0.445	14.943	0.00011**	5.582
2	Age (old)	0.053	0.014	13.637	0.00022**	1.055
3	A/G ratio (decrease)	0.723	0.336	4.646	0.03113**	2.061
4	General fatigue	-0.652	0.304	4.593	0.03211**	0.521
5	Arthralgia	0.633	0.299	4.478	0.03433**	1.884
6	Gender (female)	0.615	0.334	3.390	0.06558*	1.850
7	Pigmentation of the right buccal mucosa (band-like)	2.596	1.422	3.332	0.06793*	13.416

* *P *< 0.10, ** *P *< 0.05

PCB blood level as an objective variable, 2,3,4,7,8-PeCDF blood concentration, sputum, age, female gender, past history of pigmentation and acneform eruption, toe nail pigmentation, hepatomegaly, headache, cheesy secretion from meibomian glands, total bilirubin, and general fatigue showed less than a 0.05 level of significance. When 2,3,4,7,8-PeCDF blood concentration was excluded from the explanatory variables, age, sputum, past history of pigmentation, total bilirubin, PCQ blood concentration, toe nail pigmentation, arthralgia, presence of a chief dental complaint, headache, and cheesy secretion from meibomian glands were significantly correlated with PCB blood concentrations.

In contrast, when 49 factors were extracted by the principal component analysis (2,3,4,7,8-PeCDF and PCQ as objective variables, PCB blood concentration as an explanatory variable), items which showed less than a 0.05 level of significance for PCB blood concentration (explanatory variable) included 2,3,4,7,8-PeCDF blood concentration and excessive eye discharge. (Table 5)

**Table 5:** Results of logistic regression analysis (PCB blood level as an objective variable)

Explanatory variables that showed less than 0.10 level of significance when the PCB blood level (2 categories) and factors selected by the principal components analysis were used as objective variables and explanatory variables, respectively						
	Explanatory variables	β	Standard deviation	Wald	Significance probability	Exp (B)

1	2,3,4,7,8-PeCDF blood level	0.012	0.003	15.412	0.00009**	1.012
2	Sputum	-2.818	0.855	10.876	0.00097**	0.060
3	Age (old)	0.129	0.039	10.834	0.00100**	1.137
4	Past history of pigmentation	-3.832	1.167	10.783	0.00102**	0.022
5	Gender (female)	-1.986	0.693	8.220	0.00414**	0.137
6	Past history of acneform eruptions	2.785	1.023	7.409	0.00649**	16.202
7	Pigmentation (toe nails)	-1.907	0.750	6.472	0.01096**	0.149
8	Hepatomegaly	-7.627	3.134	5.920	0.01497**	0.000
9	Headache	-1.554	0.704	4.871	0.02732**	0.211
10	Cheesy secretion from meibomian glands	2.623	1.248	4.414	0.03564**	13.773
11	Total bilirubin (increase)	-1.611	0.770	4.372	0.03654**	0.200
12	General fatigue	1.374	0.676	4.138	0.04194**	3.951

Explanatory variables which showed less than 0.10 level of significance when 2,3,4,7,8-PeCDF blood level was excluded from the explanatory variables in the above analysis						

	Explanatory variables	β	Standard deviation	Wald	Significance probability	Exp (B)

1	Age (old)	0.115	0.033	11.920	0.00056**	1.121
2	Sputum	-2.040	0.663	9.473	0.00208**	0.130
3	Past history of pigmentation	-2.432	0.958	6.440	0.01116**	0.088
4	Total bilirubin (decrease)	-1.701	0.686	6.145	0.01318**	0.182
5	PCQ blood level	1.142	0.480	5.654	0.01742**	3.134
6	Pigmentation (toe nails)	-1.418	0.621	5.213	0.02242**	0.242
7	Arthralgia	-4.886	2.357	4.299	0.03814**	0.008
8	Presence of a chief dental complaint	1.544	0.748	4.263	0.03896**	4.682
9	Headache	-1.276	0.624	4.179	0.04093**	0.279
10	Cheesy secretion from meibomian glands	2.616	1.291	4.110	0.04264**	13.682
11	Past history of acneform eruptions	1.650	0.853	3.743	0.05304*	5.205
12	Black comedones	-3.569	2.086	2.926	0.08716*	0.028
13	Mean corpuscular volume (increase)	-0.080	0.047	2.847	0.09154*	0.923
14	Urinalysis protein (increase)	-0.619	0.372	2.771	0.09600*	0.539

* *P *< 0.10, ** *P *< 0.05

PCQ blood concentration as an objective variable, tooth pigmentation, arthralgia, γ-GTP, total bilirubin, cheesy secretion from meibomian glands, general fatigue, total cholesterol, toe nail pigmentation, female gender, and oral mucosa pigmentation all showed less than a 0.05 level of significance. When 2,3,4,7,8-PeCDF blood concentration was excluded from the explanatory variables, past history of pigmentation, tooth pigmentation, PCB blood concentrations, acneform eruption, abdominal pain, pigmentation, and total cholesterol were significantly correlated with PCQ blood concentrations. (Table 6)

**Table 6:** Results of logistic regression analysis(PCQ blood levelas an objective variable)

Explanatory variables which showed less than 0.10 level of significance when PCQ blood level (2 categories) and the factors selected by the principal components analysis were used as objective variables and explanatory variables, respectively.						
	Explanatory variables	β	Standard deviation	Wald	Significance probability	Exp (B)

1	2,3,4,7,8-PeCDF blood level	0.040	0.010	16.276	0.00005**	1.041
2	Tooth pigmentation	6.737	2.073	10.564	0.00115**	843.324
3	Arthralgia	-3.082	1.138	7.337	0.00675**	0.046
4	γ-GTP (increase)	-0.065	0.024	7.132	0.00757**	0.937
5	Total bilirubin (decrease)	3.282	1.384	5.626	0.01769**	26.621
6	Cheesy secretion from meibomian glands	-7.612	3.230	5.554	0.01844**	0.000
7	General fatigue	2.983	1.273	5.497	0.01905**	19.756
8	Total cholesterol (increase)	0.042	0.018	5.260	0.02182**	1.043
9	Pigmentation (toe nails)	-3.974	1.906	4.348	0.03705**	0.019
10	Gender (female)	-2.227	1.111	4.015	0.04509**	0.108
11	Pigmentation of the right buccal mucosa (band-like)	-7.584	3.846	3.889	0.04860**	0.001
12	Acneform eruptions (other sites)	8.124	4.436	3.354	0.06705*	3372.941
13	Headache	-1.901	1.116	2.903	0.08842*	0.149

Explanatory variables which showed less than 0.10 level of significance when 2,3,4,7,8-PeCDF blood level was excluded from the explanatory variables in the above analysis						

	Explanatory variables	β	Standard deviation	Wald	Significance probability	Exp (B)

1	Past history of pigmentation	3.117	0.989	9.932	0.00162**	22.588
2	Tooth pigmentation	3.929	1.260	9.727	0.00182**	50.869
3	PCB blood level	0.437	0.156	7.842	0.00510**	1.547
4	Acneform eruptions (trunk)	-3.260	1.297	6.318	0.01195**	0.038
5	Abdominal pain	-1.779	0.781	5.184	0.02280**	0.169
6	Pigmentation (face)	4.678	2.105	4.937	0.02629**	107.516
7	Total cholesterol (increase)	0.021	0.010	4.310	0.03790**	1.021
8	Acneform eruptions (other sites)	3.613	2.003	3.252	0.07132*	37.059
9	Pigmentation of the upper lip (patchy)	3.161	1.830	2.984	0.08407*	23.590
10	γ-GTP (increase)	-0.021	0.013	2.721	0.09901*	0.980

* *P *< 0.10, ** *P *< 0.05

## Discussion

PCBs, PCQs and PCDFs are known as the causative agents of Yusho. The results from this study show that, the concentrations of 2,3,4,7,8-PeCDF, PCB and PCQ in blood were strongly related. The blood concentrations of 2,3,4,7,8-PeCDF, PCB and PCQ tended to correlate with older age, as adult victims were considered to have eaten greater amounts of the contaminated oil compared with child victims when the contaminated oil was available in shops in 1968. The blood concentrations of 2,3,4,7,8-PeCDF, PCB and PCQ also tended to correlate with female gender. This may be attributed to the fact that these chlorinated compounds are highly lipophilic and accumulate in adipose tissue [3]. Females who have more adipose tissue may have thus accumulated more 2,3,4,7,8-PeCDF, PCB and PCQ.

In our study, the blood concentrations of 2,3,4,7,8-PeCDF, PCB and PCQ also tended to correlate with acneform eruptions, black comedones, cutaneous and mucosal pigmentation, and hypersecretion of meibomian glands, in addition to general fatigue, headaches, cough, sputum, abdominal pain, increased serum γ-GTP, and decreased total bilirubin. These signs and symptoms are all included in the present diagnostic criteria of Yusho (Table 1). In addition to the symptoms listed in the diagnostic criteria, arthralgia was frequently correlated to 2,3,4,7,8-PeCDF, PCB and PCQ blood concentrations. Using the 2,3,4,7,8-PeCDF blood concentration as an objective variable, cases including or not including the PCB and PCQ concentrations as explanatory variables were compared. As a result, arthralgia and A/G ratio were related to the 2,3,4,7,8-PeCDF blood concentration.

Using the PCB blood concentration as an objective variable, cases including or not including the 2,3,4,7,8-PeCDF concentration as an explanatory variable were compared. As a result, PCB blood concentration was strongly related to ophthalmological symptoms.

The PCQ blood concentration was related to cutaneous, oral and ophthalmological manifestations, increased γ-GTP, and increased total cholesterol. When the 2,3,4,7,8-PeCDF blood concentration was excluded in the explanatory variables, oral pigmentation and increased total cholesterol were significantly related to PCQ blood concentration. The biochemical adverse effect of PCQ has been reported to include increased triacylglycerol concentration [20]. However, based on the results of this study, total cholesterol concentration, one of the markers of lipid metabolism such as triacylglycerol, was related to PCQ blood concentration.

Like Kanemi Yusho, Taiwan Yucheng, a health hazard caused by PCB or PCDFs, has been reported to have a high incidence of symptoms of chloracne, goiter, arthritis, and anemia [21,22]. Chloracne and arthritis are considered e symptoms common to Yusho and Taiwan Yucheng. Health hazards caused by 2,3,7,8-tetrachlorodibenzo-p-dioxin (TCDD) in the Seveso (Italy) event have also been studied. In a death survey, conducted 20–25 years after the Seveso (Italy) event, a high incidence of deaths due to cancer, circulatory disease, chronic obstructive pulmonary disease (COPD), and diabetes mellitus was reported [23,24]. It thus seems necessary to examine the presence of a relationship between Yusho and COPD in the future, since cough, sputum, and bursitis, included in the Yusho criteria, are also symptoms seen in COPD.

Cutaneous, mucosal and ophthalmological manifestations, related to the blood concentrations of 2,3,4,7,8-PeCDF, PCB and PCQ in this study, were considered characteristic of Yusho and were included in the diagnostic criteria.

## Conclusion

Although 35 years have passed since the occurrence of Yusho, the 2,3,4,7,8-PeCDF blood concentration appeared related to the PCQ and PCB blood concentrations, arthralgia and A/G ratio; The blood PCB concentration was strongly related to ophthalmological symptoms; while PCQ blood concentration was related to total cholesterol. These findings suggest that the co-contaminants may affect other functions than those originally associated with Yusho.

## Abbreviations

PCBs: polychlorinated biphenyls; PCQs: polychlorinated quarterphenyls; PCDFs: polychlorinated dibenzofurans; TCDD: 2,3,7,8-tetrachlorodibenzo-p-dioxin.

## Competing interests

The authors declare that they have no competing interests.

## Authors' contributions

YK designed the study and drafted the manuscript. SM designed the data analysis, analyzed data, and assisted manuscript drafting. SK assisted manuscript drafting. BT assisted designing of data analysis. NF, SS and HU collected data. MF designed the whole study and assisted manuscript drafting. All the authors, except TI, reviewed the final manuscript and all authors read and approved the final manuscript.
